# Supplementary material for: Are carbon emissions trading and green financial instruments synergistic? -Comprehensive quantitative research based on content analysis
Source: PLoS One. 2024 Mar 7;19(3):e0298601. doi: 10.1371/journal.pone.0298601 (PMC10919601; doi:10.1371/journal.pone.0298601)
Supplement: S1 Data — (ZIP) [file pone.0298601.s001.zip › Data/地级市Z_F工作报告.zip!/╔┬╬≈/╔╠┬σ╩╨/└┤╫╘╣1⁄2╓┌║┼í╛┬φ┐╦╩2╛▌═°í┐_2016.docx]

各位代表：

现在，我代表市人民政府向大会作工作报告，请各位代表连同《商洛市国民经济和社会发展第十三个五年规划纲要（草案）》一并审议，并请市政协委员及列席人员提出意见。

一、2015年和“十二五”工作回顾

2015年，面对商洛建市以来最复杂的发展环境、最严峻的经济下行压力，在市委坚强领导下，我们迎难而上、奋力拼搏，干成了一批打基础、利长远的大事，攻克了不少啃骨头、拔钉子的难事，办成了诸多惠民生、暖民心的实事，基本完成市三届人大五次会议确定的各项任务。全年实现生产总值621.83亿元、增长11.2%，增速位居全省第三；财政总收入45.02亿元,地方财政收入31.79亿元，同口径分别增长10%和12.8%；城乡居民收入达到26896元和7732元，分别增长8.77%和9.91%，增速均居全省第一。

（一）针对下行压力精准施策，经济持续稳定增长。把促投资稳增长放在首要位置，先后争取中省资金143.2亿元、政府债券56.9亿元，落实政策性银行贷款72.99亿元，160个市级重点项目强力推进，累计抢开促投资项目246个，电动客车、恒瑞肝素钠研发等45个项目基本建成，商洛电厂、山柞高速、中药材战略储备库等一批重大项目启动建设，全年完成固定资产投资767.69亿元、增长22.8%。把企业稳产促销作为重中之重，出台工业稳增长23条政策措施，扶持有销路有效益的企业扩大生产、开拓市场，完成规模以上工业增加值206.14亿元、增长18%。把农业园区建设作为主要抓手，大力发展特色现代农业，省级现代农业园区增加到19个，粮食生产实现“八连丰”，农产品质量和效益显著提升，成功创建为“国家农产品质量安全示范市”。把扩大消费需求作为主攻方向，旅游消费持续攀升，新兴消费蓬勃兴起，网络消费成为新热点，实现社会消费品零售总额154.66亿元、增长13%。经济的稳定增长，既保证了就业和群众增收，又拓展了调结构的空间。

（二）围绕提质增效优化结构，发展方式加快转变。坚持依托资源、依靠科技，促传统产业升级，促新兴产业发展，产业结构在逆境中加速调整，新技术新业态不断涌现，新的增长点加快孕育并破茧而出。绿色食品、生物医药产值分别增长42%和25.8%，新材料、先进制造业产值分别增长25.1%和26%，战略性新兴产业增加值增长16%，矿业占比下降3个百分点。以旅游为重点提升现代服务业层次，成功举办2015秦岭生态旅游节和环秦岭公路自行车赛，金丝峡成为陕南首家5A级景区，旅游总收入达到174.9亿元、增长14.1%；“互联网+”、电子商务、物流快递等新业态快速发展，服务业增加值增长10.7%，占GDP比重达到33.23%，增速创三年来新高。多点支撑、多元带动的产业格局正在形成，在应对经济下行中发挥了重要作用。

（三）立足城乡统筹协调发展，城镇建设成效明显。坚持城为民建、市为民享，“一体两翼”中心城市格局初具雏形，87个城建项目完成投资68.3亿元，“十大城建”、“十大棚改”在艰难中稳步推进，环城北路建成通车，商丹、商洛两条城市干线建设进展顺利，南秦新区、蟒岭绿道建设全面启动，市文化艺术中心、新建商洛中学等大型公共工程加快推进，城市功能日趋完善，聚集作用日益显现。城镇建设完成投资34.1亿元，县城扩容提质进程加快，县域经济实力大幅提升，沙河子省级重点示范镇建设走在全省前列，棣花、凤凰、漫川关、云盖寺等特色小镇享誉全省。“美丽乡村”建设取得突破性进展，集中打造了朱家湾、前店子等一批各具特色的美丽乡村示范村，成为幸福商洛的新名片。

（四）狠抓污染治理保护环境，生态质量显著提升。系统推进绿色低碳循环发展，10大循环产业链全部建成，16个标志性工程基本建成。实施丹江等流域污染防治项目105个，丹江出境断面水质稳定达标。强化秦岭生态保护，狠抓农村环境连片综合整治，加强面源污染治理，统筹实施造林绿化、退耕还林、湿地恢复保护、小流域综合治理，完成植树造林32.6万亩，治理水土流失700平方公里。严格落实节能减排减碳措施，万元GDP能耗下降3.1％，中心城区222台燃煤锅炉全部拆改到位，淘汰“黄标车”662辆，四项主要污染物减排任务超额完成。“治污降霾”成为全省标杆，全年新标准良好以上天数306天，呵护了全省最美蓝天，“商洛蓝”成为2015网络热词。

（五）强推改革创新扩大开放，发展活力充分释放。政府机构改革全面完成，行政编制精简10%，镇村综合改革经验在全省推广。简政放权和商事制度改革稳步推进，取消下放行政审批事项151项，精简率41.1%，新增各类市场主体1.4万户、增长24.8%，非公有制经济占比达到53.1%。公车改革稳步实施，投融资和国企改革不断深化，融资性担保公司和小额贷款公司发展到18家，市城投、交投等五大国有企业融资保障能力显著增强。陆路货运口岸及省级出口基地加快建设，完成外贸进出口总额2.93亿美元，招商引资到位资金430.9亿元、增长18%。创新创业风起潮涌，电子商务异军突起，阿里巴巴“农村淘宝”走进商洛，山阳、商南成为省级电子商务示范县，全市电商企业达到383家，交易额突破20亿元，电子商务成为转方式、调结构、促消费的重要引擎。

（六）落实兜底补短各项政策，民生民本持续改善。在财政收入增速放缓、支出压力加大的情况下，全年民生支出149亿元、增长13%，保障能力持续提高。深入开展省级创业型城市创建，以创业带动就业，新增城镇就业1.85万人，农村劳动力转移就业54.7万人。社保体系不断完善，机关事业单位养老并轨全面完成，企业退休人员养老金实现“十一连增”，城镇居民医保和新农合人均财政补助分别达到400元和500元，新农合参合率100%。“双包双促”精准扶贫深入开展，移民搬迁、棚户区改造和保障房建设走在全省前列。教育质量稳步提升，高考二本以上上线率连续8年位居全省第一。医药卫生体制改革深入推进，分级诊疗制度启动实施。文体惠民工程加快推进，体育器材配送实现镇办全覆盖，以《带灯》为代表的一批精品剧目亮相全国舞台。

2015年，我们不断加强政府自身建设，严格落实全面从严治党各项部署，扎实开展“三严三实”专题教育，认真贯彻八项规定精神，坚决纠正“四风”突出问题，全面实行财政预决算和“三公”经费公开，全市“三公”经费支出下降13%，查处政府系统违纪人员192人。始终坚持依法行政，全面完成“六五”普法，健全依法决策机制，主动接受市人大依法监督，自觉接受市政协民主监督，办理人大代表建议273件、政协提案285件，向人大报告专项工作30项，接受市人大执法检查2次、专题询问1次，参与市政协月度协商6次。全面加强安全生产、信访维稳、社会治安和应急管理等工作，政府社会治理能力不断提升。同时，国防动员、民族、宗教、侨务、对台、地震、气象、档案、统计、人防、水文、方志、无线电、工商联、妇女儿童、红十字会等领域的工作也取得了新的成绩。

各位代表，2015年主要目标任务的完成，标志着“十二五”胜利收官。“十二五”确定的主要指标，16项超额完成，4项全面完成，4项基本完成，为全面建成小康社会奠定了坚实基础。回首过去的五年，我们经受住了考验，付出了艰辛，创造了辉煌。

——综合实力大幅跃升。生产总值年均增长12.9%，高于全省1.8个百分点，总量是2010年的2.2倍，人均生产总值突破4000美元；财政总收入、地方财政收入年均增长16.2%和21.5%，总量是2010年的2.1倍和2.6倍。经济规模和质量实现“双提升”，支撑发展和保障民生的能力显著增强。

——发展基础更加坚实。固定资产投资是上个五年的2.8倍，在交通、水利、电力、环保和产业发展等领域，兴建了一批补短板、蓄势能的重大项目。“五件大事”取得重大突破，西武客专列入国家“十三五”规划，商丹园区创建省级高新技术开发区通过评审，镇安抽水蓄能电站辅助性工程先行启动。西康、西宁铁路复线建成运营，高速公路通车里程达到409公里，在全省率先实现县县通高速，昔日交通短板已成为竞争新优势。

——工业增速领跑全省。园区成为工业转型的重要载体，以商丹园区为骨干、15个省级重点工业集中区为支撑，促进了企业集中、要素集聚、产业集群。现代材料、绿色食品、生物医药产值占全部工业总产值的85.9%，新能源汽车、电子信息、智能制造等新兴产业加速成长。规模以上工业企业达到230户，较2010年增加89户，总产值、增加值年均分别增长35.7%和25.4%，增速连续五年位居全省第一。

——城乡面貌焕然一新。“一体两翼”中心城市产城融合迈出坚实步伐，城市规划区建成面积达到49.1平方公里。县城、重点镇和美丽乡村建设亮点纷呈，省级卫生城市和园林城市创建实现全覆盖，2个县创建为“国家卫生县城”，12个镇进入全国重点镇，建成新型城乡社区690个、美丽乡村22个，35万农村居民成为新市民，全市城镇化率达到49.5%。城乡面貌的巨大变化，更加增强了全市人民加快发展的自信心。

——创新潜能有效释放。创新型城市创建全面启动，实施省部级科技专项150项，荣获省级以上科技奖15项，技术交易额2.2亿元、专利授权量883件，分别是五年前的448倍和5倍，科技对经济增长贡献率达到53%。积极顺应“互联网+”发展趋势，推进大众创业万众创新，建成科技园、农民工返乡创业园等众创平台12个，市级创业孵化基地7个，入住孵化企业237家。“双创”点燃“草根”创业的梦想，为企业插上腾飞的翅膀，成为催生新动能的重要力量。

——最美商洛魅力彰显。强力推进精品景区、文化旅游名镇、美丽乡村建设，发展全域旅游，促进旅游联盟。全市建成3A级以上景区21家，创建省级旅游示范县2个、省级特色旅游名镇7个、省级乡村旅游示范村17个。成功打造了“一节一赛”品牌，游客人数和收入年均增长18.5%和26.6%。“秦岭最美是商洛”唱响全国，商洛的知名度和影响力大幅提升。

——生态成为最靓名片。循环发展成效显著，成功跻身国家首批循环经济示范市、国家生态文明示范工程试点市。生态建养造福百姓，在全省率先启动丹江等流域污染防治，流域治理“河长制”经验在全省推广，森林覆盖率达到66.5%，较2010年提高4.2个百分点，涵养了“一江清水”，呵护了“最美蓝天”，空气质量五年稳居全省第一。清洁的水质、翠绿的青山、最美的蓝天，成为老百姓幸福生活最直观的体现。

——民生福祉显著改善。“两个80%”全面落实，就业和社保体系持续完善，农民收入年均增速跑赢GDP。“双包双促”精准扶贫经验在全省推广，46.9万贫困人口稳定脱贫，306名贫困家庭子女免费就读商洛职院。6.9万户居民通过安居工程迁入新居，9.2万户33.6万人依靠移民搬迁告别深山。平安建设成果丰硕，连续五年荣获“全省社会治安综合治理优秀市”，公众安全感稳居全省前列，全市人民生活得更有尊严、更加自豪。

各位代表，过去五年，是我们科学应对挑战，勇闯难关、锐意进取、全面深化改革的五年；是我们积极适应经济新常态，优化结构、转变方式、全面提质增效的五年；是我们紧跟全国全省步伐，缩小差距、奋力追赶、奠定全面小康基础的五年。五年的奋斗充满艰辛，五年的成就令人欣喜，五年的经验弥足珍贵。我们深深体会到：无论形势多么严峻、环境多么复杂，必须牢牢把握科学发展这个第一要务，抓住用好机遇，发挥自身优势，扬长避短，加快发展；必须牢牢把握改革创新这个核心动力，破除体制机制障碍，打破思维定势，厚植优势，抢占先机；必须牢牢把握团结和谐这个制胜法宝，巩固心齐气顺劲足的良好政治生态，低调务实不张扬，埋头苦干，合力攻坚；必须牢牢把握改善民生这个根本目的，突出保基本、补短板、兜底线，让改革发展成果更多更公平地惠及全市人民。

各位代表，过去五年我们取得的成绩，是省委、省政府和市委坚强领导的结果，是市人大依法监督和市政协民主监督的结果，是全市上下万众一心、奋勇拼搏的结果。在此，我谨代表市人民政府，向全市人民、驻商部队指战员、武警官兵和公安民警致以崇高的敬意！向大力支持政府工作的各位代表、各位委员和社会各界人士表示衷心的感谢!

在肯定成绩的同时,我们也清醒地看到，由于受多种因素影响，个别预期性指标未达到计划进度，我市发展仍面临诸多困难和问题：贫困人口多、群众收入低，如期脱贫摘帽、实现全面小康的任务极为艰巨；经济结构不优、产业层次不高、创新能力不强，调结构、转方式仍是我们的长期任务；各类人才匮乏，尤其是懂经济、会管理人才短缺，认识、把握、引领新常态的能力有待进一步提高；财税增长放缓和刚性支出增多的矛盾日益凸显，财政收支平衡困难加大；市场调控、社会管理和公共服务能力有待进一步加强，持续改进作风、建设法治政府仍需我们不断努力。对此，我们必须高度重视，采取有效措施，认真加以解决。

二、“十三五”发展的总体思路和目标任务

“十三五”是全面建成小康社会的决胜阶段。当前，世界经济延续疲弱复苏态势，国内经济将进入“L”型发展阶段，有效需求乏力和有效供给不足并存，但经济发展长期向好的基本面没有改变。党的十八届五中全会、中央经济工作会明确提出“五大发展理念”、“五大政策支柱”、“五大重点任务”和“十个更加注重”，为我们廓清了思路、指明了方向。“十三五”时期，国家将深入推进“一带一路”战略，加强供给侧结构性改革，打赢脱贫攻坚战，加快革命老区振兴发展；省委、省政府务实推进丝绸之路经济带新起点建设，明确支持西商一体化发展，为我们带来新的重大机遇。特别是经过多年探索和实践，我市发展思路更加清晰，发展基础更加牢固，产业支撑更加强劲，全面建成小康社会的步伐更加坚定。

未来五年发展的总体思路是：全面贯彻党的十八大和十八届三中、四中、五中全会精神，以邓小平理论、“三个代表”重要思想、科学发展观为指导，深入贯彻习近平总书记系列重要讲话精神，以“五位一体”总体布局和“四个全面”战略布局为统领，坚持创新、协调、绿色、开放、共享发展理念，坚持追赶超越、绿色循环发展主线，扎实推进供给侧结构性改革，全力打好现代工业、现代特色农业、新型城镇化、精准脱贫四个攻坚战，依托大西安、建设大商洛、打造大品牌，倾力建设秦岭休闲之都、丝路产业新城，确保如期全面建成小康社会，开创美丽幸福商洛建设新局面。

未来五年的主要目标是：聚焦实现全面小康，建设美丽幸福新商洛，强力推进西商一体化发展，努力实现“四个翻两番”和“五个再提升”。即：生产总值、城乡居民收入、财政收入分别比2010年翻两番，固定资产投资累计比“十一五”翻两番。生产总值突破千亿元大关，达到1150亿元，年均增长10%左右；城镇居民收入达到4.5万元，农村居民收入达到1.45万元，年均分别增长11%和13.5%左右；财政总收入、地方财政收入达到85亿元和55亿元，年均分别增长13.5%和12%左右。固定资产投资五年累计达到6000亿元左右，年均增长16%以上。实现产业结构、内生动力、公共服务、生态质量、开放水平“五个再提升”，推进全市经济保持中高速增长、产业迈向中高端水平。

未来五年的主要任务是：

坚持创新发展，构建现代产业新体系。把创新作为第一动力，培育发展新动能，构建产业新体系。深入实施创新驱动战略，以科技创新带动产品、管理和商业模式创新，不断优化产业结构，大力发展新兴产业，推进工业转型升级，加快发展现代特色农业，着力推进全域旅游，培育壮大新型服务业态，打造“334”十大优势产业集群。发挥创新激励经济增长的乘数效应，积极推进大众创业万众创新，率先在陕南建成创新型城市，商丹园区建成国家高新技术产业开发区。到2020年，三次产业结构调整为10:50:40，消费对经济增长贡献率达到40%以上，科技进步贡献率提高到57%。

坚持协调发展，推动城镇建设新跨越。把协调作为内在要求，优化区域发展布局，构建新型城镇体系。以实施西商一体化发展为引领，做强“一体两翼”中心城市，推进商丹园区创新发展，支持洛河园区扩容提质，倾力打造丹江、洛河两大产业长廊。坚持精心规划、精美建设、精细管理，支持各县发挥优势错位发展，打造精品县城，壮大县域经济。坚持因地制宜、突出特色，加快建设一批旅游文化名镇、特色产业强镇、边贸物流重镇、生态宜居新镇。坚持规划引领、产业融合，加快美丽乡村建设。围绕互联互通、平衡发展，统筹抓好交通、水利、电力、环保等重大基础设施建设。到2020年，全市城镇化率达到60%，中心城市建成区面积达到80平方公里、人口达到80万人。

坚持绿色发展，建设生态文明新家园。把绿色作为必要条件，按照“保护一江清水、呵护最美蓝天、守护秦岭绿肺”的总体要求，把生态文明建设融入经济发展全过程，率先建成国家生态文明综合改革示范区。系统推进绿色低碳循环发展，加快传统制造业、矿产建材业绿色化改造，积极推广绿色建筑，建成国家循环经济示范城市。统筹实施治污碧水、降尘护蓝、生态修复、农村清洁、节能减碳等工程，推进山水林田湖一体化治理，森林覆盖率达到69.5%，让绿色成为商洛最绚丽的色彩，让“商洛蓝”成为最普惠的民生福祉。

坚持开放发展，增强竞争发展源动力。把开放作为必由之路，主动融入“一带一路”建设，深化国际友城交流合作，推动商洛二级陆路货运口岸建成通关，着力打造内陆开放新区域。把改革作为开放的核心举措，系统推进经济、文化、社会、生态等领域改革，扎实抓好供给侧结构性改革，全面落实权力清单、责任清单、负面清单制度，为开放营造更好的环境。坚持引资、引技、引智相结合，突出绿色招商、资本招商，探索境外招商。“十三五”期间，新引进国内外500强企业20家以上，招商引资到位资金突破2500亿元。巩固和发展非公有制经济，到2020年，非公有制经济占比达到58%。

坚持共享发展，促进发展成果惠民生。把共享作为本质要求，以“双包双促”为抓手，强化政策保障，健全脱贫攻坚支撑体系，举全市之力打赢脱贫攻坚战。把移民搬迁作为治本之策，确保26.1万贫困人口通过搬迁实现脱贫。把产业脱贫作为关键之举，因地制宜发展特色产业，让群众既能脱贫又能致富。把生态补偿作为新的路径，建立横向生态补偿制度，让贫困群众从“绿色银行”中获得更多收益。把教育脱贫作为重中之重，建立从学前到大学到就业“一条龙”帮扶机制，阻断贫困代际传递。把社会保障作为兜底举措，对丧失劳动能力的贫困人口实行应保尽保。2018年商南县、柞水县脱贫摘帽，2019年商州区、洛南县、镇安县脱贫摘帽，2020年丹凤县、山阳县脱贫摘帽，全市48.9万贫困人口全部脱贫。坚持公平公正、共建共享，协调发展各项社会事业，努力让发展更有温度、幸福更有质感。

三、2016年的主要任务

今年是实施“十三五”规划的开局之年。我市发展面临的困难和压力前所未有，机遇和利好也十分难得。中央推进供给侧结构性改革，打好“三去一降一补”五大歼灭战，对我市来讲，既是重大机遇、也是严峻挑战。我们必须承受由此带来的结构调整压力、资源约束压力、减税增支压力和社会稳定压力。如何变压力为动力、化挑战为机遇，关键是在“转”字上做文章。我市发展起步晚、经济总量小，正处于“船小好调头”的最佳时期，发展短板也是后发优势。只要我们不断挖掘潜力，发挥优势，补齐短板，就完全有能力化危为机、逆势而上，实现新常态下新跨越。

我们将落实发展新理念，破解发展新难题，坚持稳中求进工作总基调，着力推进供给侧结构性改革，以首战必胜的信心和决心，以涉险攻坚的勇气和智慧，统筹推进稳增长、调结构、惠民生、防风险各项工作，切实抓好去产能、去库存、去杠杆、降成本、补短板五大任务，聚创新之力，得协调之和，享绿色之美，释开放之利，成共享之功，确保“十三五”开好局、起好步。

今年的主要预期目标是：生产总值增长10%左右，地方财政收入同口径增长10%左右，城镇登记失业率控制在3.9%以内，城乡居民收入分别增长11%和13%左右，CPI涨幅控制在3%以内。

（一）积极推进供给侧结构性改革

统筹推进“去产能”和“三个专项行动”。制定“去产能”实施方案，重点推进矿产行业减量提质增效，促其延伸链条和循环利用，寻找新的产销平衡点。积极争取中省专项奖补资金，坚决淘汰“僵尸企业”，对连续亏损且不符合结构调整方向的企业，采取资产重组、产权转让、关闭破产等方式予以“出清”，妥善安置分流人员。分类推进国企改革，优化国有资本配置，发展混合所有制企业。积极实施改善消费品供给、降本增效和制造业升级“三个专项行动”。分类施策支持有市场有效益企业扩大销售，扶持小微企业健康发展。实施质量强市战略，推动企业开发适销对路新产品。从降低交易、人工、物流、财务成本和税费、五险一金、电力价格等方面发力，清理规范中介服务，实施涉企收费目录清单管理，打好降本增效“组合拳”。

围绕市场化目标健全要素配置体系。改革科技成果使用、处置、收益管理办法，探索科技贷款风险补偿和知识产权质押融资模式，加快市级产业技术共享平台建设，充分释放创新要素活力。加强政府债务管理，整合财政专项资金，创新投入方式，通过设立产业发展基金、推行股权投资、完善财政贴息和政府购买服务，吸引更多社会资本投向产业。支持各类金融机构在商发展，建立政府、银行和担保机构合作机制，支持企业上市融资或发行债券。建立打击非法集资长效机制，提升金融服务实体经济能力。继续加大闲置土地处置力度，依法收回违规用地，盘活存量，提高效率，优先保障重大基础设施、战略性新兴产业和民生项目用地。加快形成供水、供气、环保等方面的价格市场化决定机制，对高耗能行业实施差别惩罚性和阶梯电价，全面实行居民用电、用水、用气阶梯价格。

聚焦突出问题深化“放、管、服”改革。全面公布市区县两级部门权力清单、责任清单，继续减少和规范行政许可事项，实行投资项目审批监管线上运行，完成行业协会商会行政脱钩试点。加快推进“证照分离”，建设企业信用信息共享平台，加强事中事后监管。推进工商质监、食品药品、城市管理等监督执法重心下移，落实政府属地监管责任，切实解决市场秩序、安全生产、市容市貌、社会诚信等领域的管理缺位问题。继续改进公共服务，加快市政务服务中心建设，推广“互联网+政务服务”，落实镇办村群众办事干部代办制度，优化办事流程，变“群众奔波”为“信息跑腿”，营造破束缚、汇众智、促创新、保公平的良好环境。

（二）扩大有效投资弥补发展短板

强力推进重大项目建设。抢抓国家补齐软硬基础设施短板机遇，精心策划一批重大基础设施和公共服务项目，积极争取中省资金支持，大力推广PPP模式，提高投资有效性和精准性。全年实施市级重点项目160个，确保固定资产投资增长16%以上。锲而不舍抓好“五件大事”，强力推进山柞高速、特高压直流输电、西气东输三线等重大基础设施项目，重点抓好中药材战略储备库、比亚迪电池材料、秦岭飞行小镇、盘龙二期等重大产业项目，加快实施国际医学中心、市妇保院搬迁等公益性项目。通过重大项目建设，扩大有效供给，聚积增长势能。

加速弥补城乡基础短板。突出“保基本、补缺项”，把城乡建设投入重点放在基础设施、公共服务、生态环保等公益性领域。全面推进海绵城市和地下综合管廊建设，规划建设一批人防地下停车场、商业街，拓展城市发展空间。积极推进光纤到楼入户，扩大城区公共无线网络。加快城镇生活垃圾资源化利用和无害化处理，完善县城和重点镇污水垃圾处理设施，配套发展人工湿地。坚持防水与蓄水并重，抓好桃庄水库、张坪水库、云镇水库和磨沟水库建设，完成6座病险水库除险加固，解决6万人安全饮水问题。完成干线公路改造70公里，新建县乡公路200公里。加大城网农网升级改造力度，实施输变电项目91个。

加快发展开放型经济。把招商引资作为对外开放的首要任务，瞄准国内外500强企业和京津冀、长三角产业转移，完善常态化招商机制，力争招商引资到位资金440亿元。强化政策支持和服务保障，促进非公有制经济扩量提质。加快陆路货运口岸建设，抓好海关和出入境检验检疫机构筹建，力争年内实现通关。实施外贸孵化器工程，以光伏产品、生物医药、农特产品出口为重点，培育外贸竞争新优势，促进外贸进出口稳定增长。建立健全投资环境评价机制和社会监督体系，对重大招商引资项目实行定向跟踪服务，建立来商投资企业定期回访制度，深化客商评价科股长活动，及时解决影响投资环境的突出问题。

（三）依靠创新驱动构建产业新体系

调整优化产业结构。做强工业主导产业。以“去产能”倒逼工业结构调整，突出抓好20个重点技改项目，推动现代材料产业向高端化、智能化、绿色化方向迈进，加快生物医药、绿色食品产业精细化、规模化发展，确保规模以上工业增加值增长15%左右。做精特色现代农业。坚持农林牧结合、种养加一体，推进传统农业与食品加工、旅游休闲等产业融合发展，提高农业综合效益。深入推进秦岭生态农业示范市创建，加快核桃、板栗、中药材基地品质提升，大力发展特色农业“八大件”，做实叫响“山地农产”品牌。抓好现代农业园区建设，培育新型农业经营主体。全面划定永久基本农田，建设高标准基本农田4万亩，发展节水灌溉面积4万亩，粮食总产55万吨，从今年起把粮食安全纳入区县政府年度考核。做大现代服务业。大力发展研发设计、融资租赁、仓储物流、市场营销等生产性服务业，培育物联网、云计算、大数据等新兴服务业。加快县域金融综合改革试点，扩大村镇银行县域覆盖面，推动P2P、众筹等互联网金融服务平台规范发展。

培育壮大新兴产业。“两化”融合改造传统产业。加快智能制造研发和产业化，持续抓好电动客车、工业机器人、汽车液压缸、动力电池等项目建设，壮大先进制造和电子信息产业。依托优势资源培育新兴产业。突出抓好商洛电厂、镇安抽水蓄能电站、金钱河梯级开发、分布式光伏发电等项目建设，推动清洁能源和新能源产业扩量升级。加快纳米新材料、含能产业园、商南硅科技园、钨钼产业园等项目建设，大力发展新材料产业。打造“双创”平台催生新产业。突出抓好“互联网+”创新创业孵化基地建设，支持工业园区与高校、科研院所建立“双创”孵化平台，让创客点子与市场需求快速对接，推动人才创业和产业升级。继续抓好工业园区建设，完善园区电子商务、物流配送等服务体系，着力把园区打造成新兴产业聚集区。

大力发展旅游产业。创新旅游发展理念。把旅游作为改善消费品供给的重要方向，按照“精品景区+文化旅游名镇+美丽乡村”的发展模式，以游客需求为导向，深入挖掘历史人文资源，大力发展全域旅游，推动生态旅游向观光度假、休闲体验并重转变。加快景区提档升级。务实推进大秦岭生态旅游核心区建设，支持柞水、商南建设国家全域旅游示范区，启动柞水国家旅游度假区创建，突出抓好商洛印象、商於古道·作家村、蟒岭绿道生态景区等项目建设，持续推进牛背梁、天竺山5A级景区和闯王寨等7个4A级景区创建。大力发展智慧旅游。加快旅游信息化基础网络建设，开发线上线下相结合的旅游服务产品，推动旅游定制化服务，实现4A级以上景区无线网全覆盖和美丽乡村示范村在线预订。深化“一节一赛”品牌。高标准办好2016秦岭生态旅游节、环秦岭公路自行车赛暨全国自行车锦标赛，推动旅游与文化、体育等产业融合发展，让广大游客在休闲运动中留下对商洛的美好记忆。

加快消费结构升级。培育新兴消费。围绕消费新需求增加有效供给，扩大信息消费和绿色消费，培育发展文化休闲、体育健身、养老康体、医养结合等消费业态。发展网络消费。加快农村电商服务站点建设，全面推进电子商务进农村，培育壮大“逛集网”、“大秦岭特产网”等电子商务平台，推动电商、网购、快递融合发展。激活大众消费。提高群众收入，增强消费预期，优化消费环境，从严打击制售假冒伪劣行为。结合城镇棚户区改造，规划建设一批特色美食商业街区，构建“15分钟”社区便民生活服务圈。全年社会消费品零售总额增长10%左右。

强化创新驱动发展。围绕产学研一体化打造创新平台。继续深化校（院）企合作，支持企业建立重点实验室、工程技术中心，支持商洛学院、商洛职院建立众创空间，建设大学科技园，提高协同创新能力。围绕产业方向构建全链条创新体系。组织实施多晶硅技术集成等10个科技专项，抓好14个省市科技成果中试基地建设，在数字化设计、快速成型等领域突破一批关键技术，促进成果就地转化。围绕机制创新强化政策支撑。完善支持企业技术创新的财税金融政策，改革科研评价制度，明确科研项目负责人及参与者在成果转化中所持股份比例。

（四）统筹城乡协调推进新型城镇化

全面提高城市规划管理水平。按照“一尊重、五统筹”的总体要求，以西商一体化发展为引领，加快形成总规、详规、专项规划相结合的规划体系，全面推行“多规合一”，确保城乡建设健康有序。按照生产空间集约高效、生活空间宜居舒适、生态空间山清水秀的要求，全面开展城市设计，将风道建设纳入城市规划和管理，统筹规划建设城市绿化带，县城绿地率达到35%以上，新建居住区绿地率不低于30%。创新城市治理方式，加强城市精细化、网格化、规范化管理，努力消除各种“城市病”。稳步推进城市执法体制改革，实现城市分级分层管理，构建责权明晰、服务优先、管理优化、执法规范、安全有序的城市管理体制。

推进“一体两翼”城市内涵式发展。健全市区县共建机制，推动主城区、南秦新区、商丹园区、洛河园区、商於古道文化景区“五大板块”联动发展，推进商丹同城化发展，支持洛南打造市域经济副中心，加快商丹、商洛城市干线路域经济带开发，通过道路互通、产业互融、功能互补、产城景互动，促进“一体”与“两翼”协同发展。坚持在建项目尽力而为、新建项目量力而行，实施中心城市建设项目54个，其中新建21个，年度投资61.3亿元。继续抓好“十大城建”工程，重点实施南秦新区、环城南路、老体育场改造、商帮广场会馆群、华阳老街等项目建设，确保市文化艺术中心、新建商洛中学、滨江银杏公园、商丹和商洛城市干线等项目建成投用。

抓好特色城镇和美丽乡村建设。支持各县完善市政基础设施，抓好产业园区建设，促进人口与产业协同聚集，增强县域经济发展动力，打造精品县城。坚持“宜工则工、宜游则游、宜商则商”，继续抓好重点镇和文化旅游名镇建设，推进城镇基本公共服务向常住人口全覆盖，打造特色鲜明的城镇体系。突出地域特色和商业业态培育，高标准编制美丽乡村建设规划，继续抓好8个美丽乡村示范村品质提升，启动8个美丽乡村重点村建设，打造产景融合的美丽乡村品牌。

强力推进棚户区改造提速。把今年确定为“棚户区改造提速年”，严格落实领导包抓责任，严厉打击违法建设，坚持周督查、月通报、季点评、年考核，强力推进中心城区“十大棚改”项目，全市新启动棚改项目21个，改造1.13万户，年度投资56.7亿元。把“去库存”作为稳定房地产市场的关键之举，建立购租并举住房制度，发挥政策集成效应，打通供需通道，采取“五个一批”举措，消化存量商品房。即：通过棚改货币化安置消化一批；加快移民安置房、商品房、限价房和公租房“四房打通”消化一批；放宽公积金提取条件，提高贷款额度，激活改善性住房需求消化一批；鼓励房地产企业调整营销策略，主动降价让利消化一批；引导存量商品房通过改变用途消化一批。

（五）系统推进生态建设保障绿色发展

着力构建绿色经济体系。大力发展循环经济，延伸拓展10大循环产业链，实施30个循环经济重点工程和综合利用项目。推动能源生产和消费革命，加快绿色矿山建设，支持建筑垃圾综合利用，扩大全市天然气消费总量，坚持公交优先发展，实施新能源汽车推广计划，完成中心城市电动汽车充电桩建设规划编制。加大节能减排力度，实施工业污染全面达标排放计划，推广高效节能和清洁生产技术，提高太阳能、地热能应用比重，加强工业、建筑、交通和公共机构节能，完成省上下达的节能减碳及四项减排任务。

重拳整治环境突出问题。实行“治污降霾”分季对标考核，强化特定时段重点防治，确保全年优良天数300天以上。全面完成丹江等流域污染防治任务，持续提升沿河林带、湿地、蓄滞洪区生态功能，实施水源地保护行动计划，抓好南水北调人工增雨基地建设。加大秦岭生态保护力度，从严查处违规采石和乱批乱建行为。加强土壤污染防控，抓好重金属及危化品污染防治，实施尾矿库专项整治。突出农村面源污染治理，建立农村环境综合整治长效机制。积极推进“三城”联创，加快实施25度以上陡坡地退耕还林，抓好城周绿化和绿道建设，加强小流域治理。全年造林绿化25万亩，治理水土流失700平方公里。

健全生态监督保护机制。创新资源利用政策，编制全市自然资源资产负债表，实行能源、水资源、建设用地等总量和强度双控制度，开展自然资源资产离任审计试点，全面落实生态环境损害责任追究办法，从源头上防止浪费资源、污染环境、破坏生态。推行排污权、碳排放量配额和市场交易，实施环境污染第三方治理，保护好商洛的绿水青山、蓝天白云。

（六）全力打好精准脱贫攻坚战

筑牢精准脱贫基础。按照扶持对象、项目安排、资金使用、措施到户、因村派人、脱贫成效“六个精准”要求，全面摸清贫困底数，制定脱贫攻坚规划，完善脱贫工作体系，逐级逐户逐人建立脱贫台账，确保全年脱贫13.3万人。加大扶贫资金投入力度，积极整合各类涉农资金用于脱贫攻坚。从今年起，市区县财政扶贫资金年均增长幅度不低于20%，确保扶贫资金只增不减。

创新精准脱贫措施。加快移民搬迁脱贫。按照与县城、城镇、园区“三靠近”原则，建设集中安置点91个，完成搬迁2.1万户、7.82万人，让3.4万贫困人口实现搬迁脱贫。扶持产业就业脱贫。培育壮大“一村一品”和县域经济，实施一批生态保护、旅游、光伏、电商等脱贫攻坚项目，发展特色种养和农特产品精深加工，增强贫困人口脱贫致富能力。创新生态保护脱贫。加大生态补偿和转移支付力度，吸纳有劳动能力的贫困人口成为生态保护人员，实现贫困人口就地脱贫。强化教育扶持脱贫。继续执行贫困户子女免费就读商洛职院政策，建立多层次、全覆盖的贫困学生助学补助体系。实行社保兜底脱贫，推进农村低保与扶贫“两线合一”，全面开展重特大疾病救助，防止因病致贫返贫。

夯实精准脱贫责任。夯实区县主体责任。严格落实脱贫攻坚党政“一把手”责任制，层层签订脱贫攻坚责任书，形成市县镇村齐抓脱贫的工作格局。夯实部门包扶责任。持续完善“双包双促”精准扶贫模式，充分发挥行业优势做好扶贫开发。强化脱贫任务考核。把脱贫攻坚作为单项考核内容，实行一票否决，建立健全年度脱贫攻坚报告和督查问责、考核评价机制，做到群众不脱贫、部门不脱责、干部不脱钩。

（七）以更大力度保障和改善民生

优先做好就业和社保工作。实行更加积极的创业就业政策，以创业型城市创建为抓手，重点做好引导高校毕业生到基层就业和自主创业，以及“去产能”分流人员再就业工作，新增城镇就业1.5万人，农村劳动力转移就业50万人。完善被征地农民、个体从业人员和农民工等群体参保政策，整合城乡居民基本医疗保险制度，落实城乡居民大病保险制度。完善城乡特困人员供养制度，落实困难残疾人生活补贴和重度残疾人护理补贴制度，发展多种形式的养老服务机构，加快市中心老年公寓建设，新建农村互助幸福院256个，实现县城公益性公墓全覆盖。

均衡配置公共服务资源。办好人民满意教育。全面完成二期学前教育三年行动计划，新建公办幼儿园9所；继续改善农村薄弱学校基本办学条件，加快城区中小学标准化建设，减少城镇学校大额班；优化整合高中和职业教育资源，积极筹建市职业教育集团，支持商洛学院向应用型大学转型。提升公共医疗水平。深化公立医院改革，建立现代医院管理制度，积极组建医疗集团和医疗联合体，加快县级医院和卫生机构标准化建设，继续为基层培养全科医生，推动优质医疗资源下沉；全面实施一对夫妇可生育两个孩子政策，切实落实失独家庭补助救助政策。发展文化体育事业。配合办好第十一届中国艺术节，健全五级公共文化服务体系，设立公共文化服务政府采购和资助目录，实施优秀中青年作家“百优计划”，推进移民搬迁有线电视户户通工程，加快建设区县标准足球场，保障好群众基本文化需求。

加强和创新社会治理。大力推进“七五”普法，完善公共法律服务体系。加快法制信访建设，健全诉求表达和多元化纠纷调解机制。坚持“党政同责、一岗双责、失职追责”，严格落实安全生产责任制，强化隐患排查治理。积极创建国家级食品安全示范城市，建立从生产到消费全过程食品安全追溯体系。加大平安创建力度，构建立体化社会治安防控体系，打击暴力恐怖、涉黑涉恶犯罪，确保网络和信息安全。扎实做好民族工作，依法管理宗教事务。

（八）进一步加强政府自身建设

重法治、严规范，建设法治政府。大力实施《法治政府建设实施纲要》，把政府工作全面纳入法治轨道，运用法治思维和法治方式履行政府职能。依法公开行政权责，强化行政权力监督制约，健全依法决策机制，落实重大决策合法性审查制度，推进全市行政机关法律顾问全覆盖。依法接受市人大及其常委会监督，积极落实市人大各项决议决定，认真办理审议意见和代表意见建议；大力支持市政协履行民主监督职能，继续坚持市政府市政协联席会议制度，主动参与市政协各类协商，扎实办好政协提案。丰富细化政务公开方式和内容，建立常态化监督机制，真正做到行政行为在阳光下运行。

强管理、勇担当，建设责任政府。坚持视责如山、守责如城、履责如冰，把从严管理干部和热情关心干部相结合，创新干部管理激励机制，对干部政治上激励、工作上支持、待遇上保障、心理上关怀，让干部安心、安身、安业，促使广大干部心情舒畅、充满信心，积极作为、敢于担当。建立合理容错机制，把干部在推进改革中缺乏经验、先行先试出现的失误和错误，同明知故犯的违纪违法行为区分开来；把上级尚无明确限制的探索性试验中的失误和错误，同上级明令禁止后依然我行我素的违纪违法行为区分开来；把为推动发展的无意过失，同为谋取私利的违纪违法行为区分开来，保护作风正派、敢抓敢管的干部，最大限度调动广大干部的积极性、主动性和创造性，激励他们担好深化改革之责，履好推动发展之责，尽好服务群众之责。

重实干、抓执行，建设效能政府。把务实重干作为政府工作的基本功，坚持问题导向、底线思维，健全和完善绩效管理、首问负责、限期办结、跟踪督办、考核评价和责任追究等制度，打通决策落地“中梗阻”。狠抓政府执行力建设，持续完善重点工作推进机制，实行清单式管理全覆盖，让广大干部心中都有“时间表”，眼前都有“施工图”，肩上扛着“任务书”。加大治庸治懒治散力度，切实解决能力不足而“不能为”、动力不足而“不想为”、担当不足而“不敢为”等“为官不为”问题，诚于谋事、善于处事、勇于担事、踏实做事，确保各项工作高质量推进、高标准完成。

守纪律、讲规矩，建设廉洁政府。始终把纪律和规矩挺在前面，认真落实“一岗双责”主体责任，进一步加强党风廉政建设，严肃查处各种违纪违法行为。持之以恒落实中央八项规定精神，巩固“三严三实”专题教育成果，推动政风民风向上向善。牢固树立过“紧日子”思想，建立厉行节约长效机制，确保“三公”经费只减不增。不断强化行政效能监察，加快推进审计监督全覆盖，重点抓好财政资金分配使用、国有资产监管、政府投资、公共工程建设等领域的监督检查。严格执行廉洁自律准则和纪律处分条例，坚持高标准和守底线相结合，扎紧制度笼子，消除寻租空间，铲除腐败土壤，营造风清气正的政治生态。

各位代表，商洛的发展离不开驻商部队和武警官兵长期的支持与奉献。我们将坚决拥护和支持国防军队全面深化改革，扎实做好国防动员、兵员征集、民兵训练、人民防空工作，满腔热情解决好军转干部、复退军人和优抚对象的实际困难，不断巩固和发展军政军民团结的大好局面。

各位代表，全面小康宏伟蓝图鼓舞人心，幸福商洛发展愿景催人奋进。让我们紧密团结在以习近平同志为总书记的党中央周围，高举中国特色社会主义伟大旗帜，在省委、省政府和市委的坚强领导下，克难攻坚，锐意进取，努力实现“十三五”良好开局，在新的发展征程中谱写商洛华彩篇章！
